# A Plant Disease Recognition Method Based on Fusion of Images and Graph Structure Text

**DOI:** 10.3389/fpls.2021.731688

**Published:** 2022-01-14

**Authors:** Chunshan Wang, Ji Zhou, Yan Zhang, Huarui Wu, Chunjiang Zhao, Guifa Teng, Jiuxi Li

**Affiliations:** ^1^National Engineering Research Center for Information Technology in Agriculture, Beijing, China; ^2^School of Information Science and Technology, Hebei Agricultural University, Baoding, China; ^3^Beijing Research Center for Information Technology in Agriculture, Beijing, China; ^4^Hebei Key Laboratory of Agricultural Big Data, Baoding, China; ^5^School of Mechanical and Electrical Engineering, Hebei Agricultural University, Baoding, China

**Keywords:** disease recognition, graph convolutional neural network, text recognition, robustness, fusion

## Abstract

The disease image recognition models based on deep learning have achieved relative success under limited and restricted conditions, but such models are generally subjected to the shortcoming of weak robustness. The model accuracy would decrease obviously when recognizing disease images with complex backgrounds under field conditions. Moreover, most of the models based on deep learning only involve characterization learning on visual information in the image form, while the expression of other modal information rather than the image form is often ignored. The present study targeted the main invasive diseases in tomato and cucumber as the research object. Firstly, in response to the problem of weak robustness, a feature decomposition and recombination method was proposed to allow the model to learn image features at different granularities so as to accurately recognize different test images. Secondly, by extracting the disease feature words from the disease text description information composed of continuous vectors and recombining them into the disease graph structure text, the graph convolutional neural network (GCN) was then applied for feature learning. Finally, a vegetable disease recognition model based on the fusion of images and graph structure text was constructed. The results show that the recognition accuracy, precision, sensitivity, and specificity of the proposed model were 97.62, 92.81, 98.54, and 93.57%, respectively. This study improved the model robustness to a certain extent, and provides ideas and references for the research on the fusion method of image information and graph structure information in disease recognition.

## Introduction

Diseases, as one of the main factors affecting the growth of crops, can cause an average annual loss of crop yield up to more than 10%. Diseases not only directly lead to the loss of crop yield, but also severely affect the quality of agricultural products and even cause food safety incidents. Therefore, automatic recognition of crop diseases plays a significant role in diagnosing the disease type as early as possible, making correct control decisions and minimizing yield loss. Meanwhile, automatic disease recognition can also help mitigate the environmental impact of chemical inputs, reduce production costs, decrease agricultural workers’ exposure to pesticides, and promote healthy and sustainable agricultural development.

The advancement of machine learning technology provides new opportunities for the image recognition of crop diseases. This technology has been widely utilized to recognize crop diseases in recent years (e.g., Mohanty et al., 2016; Fuentes et al., 2017; Wang et al., 2017; Ferentinos, 2018; Geetharamani and Pandian, 2019; Chen et al., 2020; Zhong and Zhao, 2020). Too et al. (2019) used DenseNet (Huang et al., 2016) for disease classification. Li Z. et al. (2020) conducted vegetable disease recognition by combining SEnet (Jie et al., 2017) with InceptionV3 (Szegedy et al., 2016). Regarding disease detection, Li J. H. et al. (2020) and Yang et al. (2020) proposed to use the Faster-RCNN target detection network to replace the artificial disease spot extraction method for the task of disease spot detection. The studies above have achieved high recognition accuracy, but it is noteworthy that, in the datasets they used (whether public datasets with simple backgrounds or self-collected datasets with complex backgrounds), the disease features were mostly concentrated in the central area of the images. Thus, although the accuracy of the deep learning model after training was relatively high on the dataset with the same disease severity, the growth state of the diseased leaves and the difference in data collection time might affect the later recognition effect. This problem was also found in most of other image recognition models (Eitrich et al., 2007; Menardi and Torelli, 2012). Therefore, improvement on the robustness of the recognition model is of great significance for practical application. Considering the impact of the locations of disease spots on the final recognition results, this study proposed a method of feature decomposition and recombination for constructing a secondary dataset. According to the difference in decomposition granularity, the diseased areas might appear randomly in any position of the image, so as to eliminate the impact of the location of disease spots on the robustness of the recognition model.

There are many modalities, such as image and text, can be used for recording and describing the features of crop diseases. Among various modalities, the RGB image modality can illustrate the visual features of the disease, which can be learned by deep convolutional neural networks; it is therefore the mainstream method of disease recognition at present. Another effective way to describe disease features is text, that is, to express the visual information in disease images in the form of text description. The advantage of the text modality is that text description is automatically focused on the key areas and features (e.g., leaves and disease spots) in the images. When describing disease features in the form of text, the knowledge graph can use structured data to perform pre-learning among different disease features, in order to simplify the learning process of text features. By fusing image information with text information to form complementary representation, it is possible to improve the performance of disease recognition. Wang et al. (2021) used the text vector form to represent non-image information of the disease, and combined with image information for joint training, which improved the utilization rate of non-image modal information. In tasks of fine-grained image recognition, Reed et al. (2016), He and Peng (2017), and He and Peng (2019) carried out image and text joint training by applying different training forms and feature expressions, which effectively solved the problem that the image modal expression was similar but the utilization rate of other modalities was weak in fine-grained image recognition. In aforementioned research works, other modal information rather than image data was mostly expressed in the form of text vectors to create the semantic relevance, while the features between categories were independent of each other, making text modeling relatively easy. However, in the field of disease recognition, there is a certain level of relevance between the information of different diseases. For example, the disease spots of cucumber downy mildew and cucumber bacterial angular leaf spot are both in shapes of polygons, and the disease spots of tomato powdery mildew and cucumber powdery mildew are both in white color. When independent text representation methods (e.g., bag-of-words model and Word2Vec) were used, the representations between different disease features were still independent of each other, making it impossible to establish connections between similar diseases. As a special data representation form, graph structure can be used to accurately describe the relationship between nodes. Therefore, compiling disease text information into graph structure information can greatly accelerate the learning process among various disease categories.

With the development and application of knowledge graph in practice, an increasing number of graph structure databases have been established. However, as knowledge graph is mostly created by human labor, its entity extraction and entity relationship extraction need to consume a lot of manpower and material resources. Thus, the graph neural network and graph convolutional neural network (GCN) (Kip and Welling, 2016; Li et al., 2018) based on graph structure were proposed, which could autonomously learn the relationship between entities in graph structure data so as to fully exert the advantages of data that is suitable for graph structure representation. Chen et al. (2019) performed image multi-label classification using GCN and modeled multi-label images with graph structure; eventually, they achieved higher recognition accuracy than other multi-label classification tasks. Yao et al. (2019) constructed a text graph structure based on text corpus by using the degree of adjacency between words and text words, and conducted GCN training on the text in the form of graph. They also achieved higher accuracy than other text classification methods. In the present study, a disease text graph structure was constructed according to the number of adjacency times between disease feature words and the overall disease description text. Then, by fusing the convolutional neural network with the GCN, a vegetable disease recognition model based on feature decomposition and recombination of images and graph structure text was proposed. The main contributions of this paper are as follows:

1. A vegetable disease recognition model with fusion of images and graph structure text was proposed, which could realize effective use of disease image information and disease description information.
2. Aiming at the shortcomings of conventional disease recognition methods such as poor image modal discrimination and low information utilization rate, the knowledge text graph structure data was used for synchronized training, which provides a knowledge reference for the image recognition process.
3. An image decomposition and recombination method was proposed, which could eliminate the impact of the location of disease spots on the recognition results and thereby improve the robustness of the model.

## Materials and Methods

### Data Acquisition

**FIGURE 1:**
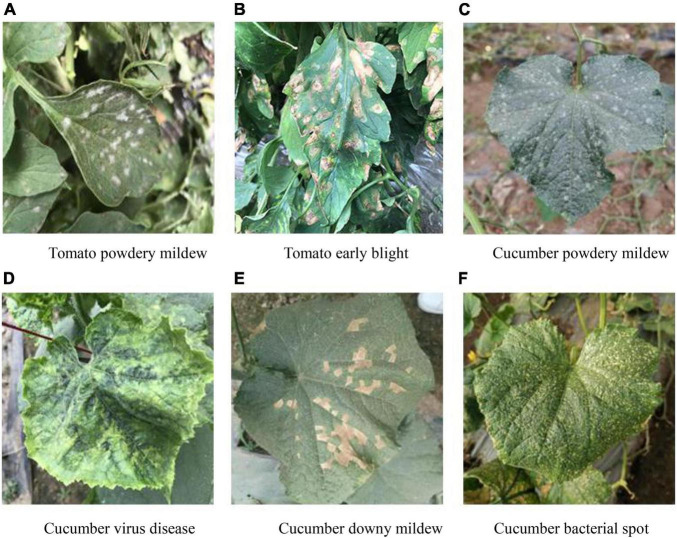
Example of dataset. **(A)** Tomato powdery mildew. **(B)** Tomato early blight. **(C)** Cucumber powdery mildew. **(D)** Cucumber virus disease. **(E)** Cucumber downy mildew. **(F)** Cucumber bacterial spot.

All the datasets used in the present study were acquired from the National Precision Agriculture Demonstration Base. The self-collected image data (covering six diseases: tomato powdery mildew, tomato early blight, cucumber powdery mildew, cucumber virus disease, cucumber downy mildew, and cucumber bacterial angular leaf spot) consisted of 1,715 leaf images, which were divided into the training set, validation set and test set according to the ratio of 7:2:1. Taking into account the impact of different lighting conditions on the image, the images were captured from June to November in three time periods: morning (7:00–8:00), noon (11:00–12:00), and evening (17:00–18:00), as shown in Figure 1. The images format is JPG and captured by mobile phones, such as Huawei, iPhone, etc. Since the images are taken from different devices, in order to unify the image size, all images are resized to 224 × 224. The original disease description text consisted of 1,715 sentences, which were manually described by five plant protection experts. The disease graph structure was then constructed according to the number of adjacency times of disease words. The original disease text is shown in Table 1. The image-text pair is used only once in the training process.

**TABLE 1:** Example of original disease description text.

Disease categories	Describe text
Tomato powdery mildew	There are two large and small white powder spots on the lower half of the surface of tomato leaves
Tomato early blight	On the front of the tomato leaf, there are several taupe spots with concentric rings
Cucumber powdery mildew	Cucumber leaves have more white, powdery spots on the underside
Cucumber virus disease	Cucumber leaves are striped on the front and wrinkled around the leaves
Cucumber downy mildew	There is a small square yellowish-green spot on the surface of a cucumber leaf
Cucumber bacterial spot	The surface of cucumber was damaged and evenly scattered with light yellow spots

### Decomposition and Recombination of Disease Features

#### Image Modality

**FIGURE 2:**
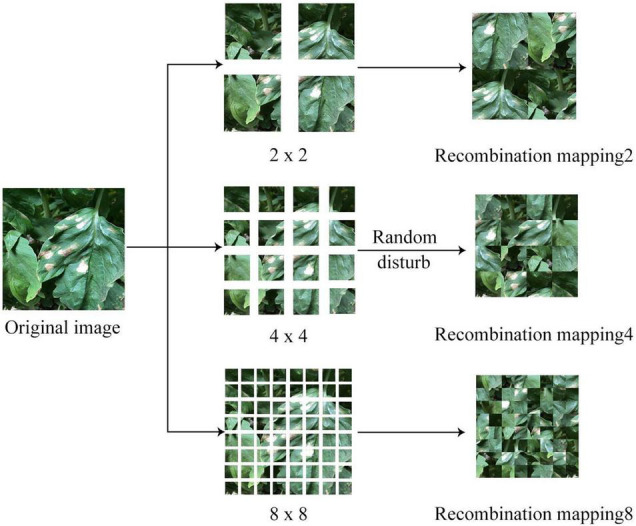
The feature decomposition and reorganization method for the image modality.

In most of the disease images, the diseased leaf occupies the central area of the image; particularly, the learner usually regards the appearance of the disease spot in the center of the image as one of the features for disease recognition during the learning process. If the diseased area appeared in a non-central position, the recognition result might be subjected to bias. Aiming at this phenomenon, a feature decomposition and recombination method was proposed, which allowed the diseased area to randomly appear at any position of the image (see Figure 2 for the process flow).

#### Text Modality

**FIGURE 3:**
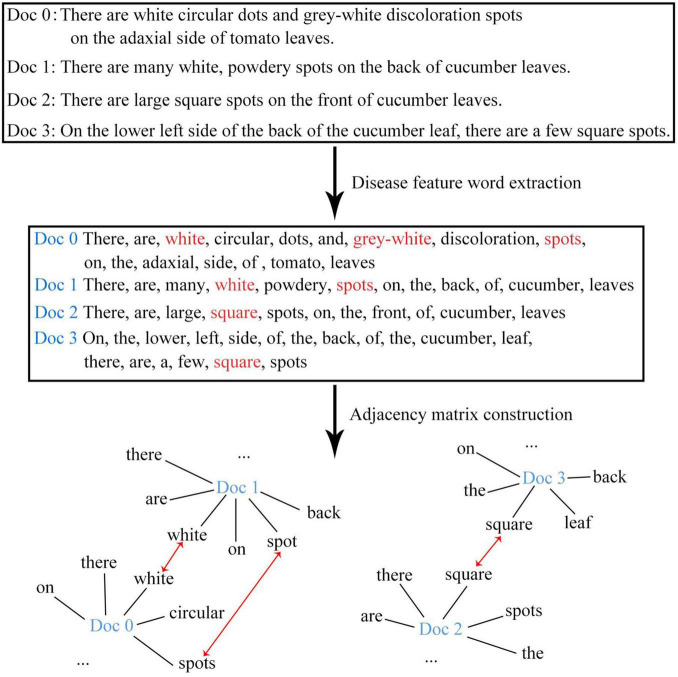
The feature decomposition and recombination method for the text modality.

The disease images in this study were all collected from the field environment. Unlike the images collected from laboratories with simple backgrounds, our images contained not only diseased leaves, but also complex background information. Moreover, the background information might change with the growth of the plant. For example, in the seedling stage of the plant, the background information was mostly soil or ground film; in the flowering stage, the background information might contain flowers; and in the fruiting stage, the background information might contain fruits. As a result, there were significant intra-category differences but insignificant inter-category differences for the same type of disease. In addition, the background information of images captured from different environments (e.g., facility environment or open-field environment) also differed greatly. When the visual information of the disease displayed by the image was being re-described in the form of text, the descriptions mainly focused on the key features of diseased leaves and disease spots (e.g., shape, color, texture, and position), while the background information was no longer included. This process managed to decouple complex background information and disease visual features to a certain extent, and thereby solved the problem of reduced recognition accuracy caused by the confusion between the backgrounds and disease features. Further, the use of natural language to describe disease features is characterized with the advantage of natural flexibility (for example, white might be described as light white, gray-white, etc.), which diversifies the disease text description and improves the robustness of the recognition model. The conventional text vectorization methods mainly use continuous vectors or dense vectors to represent text words or characters. In such a situation, the words are arranged in a continuous form without any spatial relationship. In the present study, the disease feature words in the continuous text were extracted and recombined into graph structure data that carries a spatial relationship. The workflow of feature decomposition and recombination is shown in Figure 3.

### Construction of the Vegetable Disease Recognition Model

**FIGURE 4:**
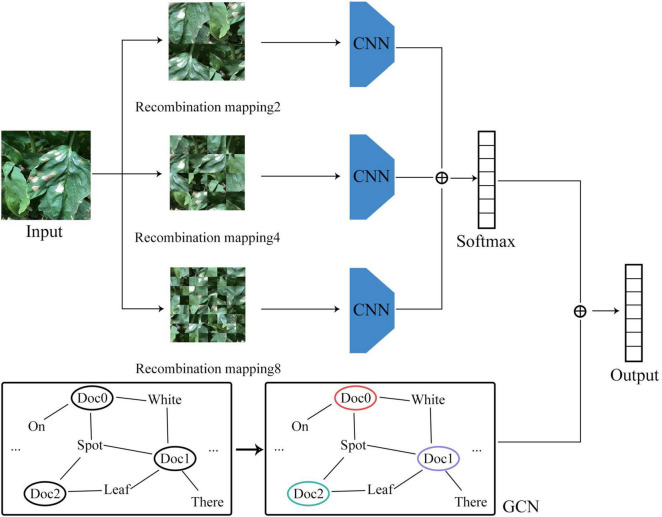
Network structure.

For the convolutional neural network model based on image data, the disease features were extracted in the form of convolution kernel sliding, while for the GCN model based on graph data, the disease features were extracted based on the relationship between the graph structure and the features of the node itself. Taking into account the correlation between the number of model parameters and model accuracy, the convolutional neural network may be set with different numbers of layers for feature extraction. But in the GCN, due to the limitation of the number of node hops, a two-layer network structure would usually be sufficient to achieve an ideal effect. The network structure of the vegetable disease recognition model constructed in this study is shown in Figure 4.

#### Image Branch

In order to mitigate the impact of the location of the diseased area on the learning process, a given original image I was randomly segmented and disarranged at different granularities. The specific rules are shown in Eq. 1.


(1) $$\tilde{\text{I}}=\text{F}{\left(I/s\right)}_{\text{s}=2,4,8}$$

where F(⋅) refers to the recombination function after random disarrangement; *S* refers to the granularity of image segmentation. In this study, three granularities were set, namely 2, 4, and 8, and the corresponding number of image blocks after segmentation was 4, 16, and 64, respectively. As the granularity continued to increase, the level of image confusion would gradually increase.

After obtaining image blocks at different granularities upon segmentation, disarrangement, and recombination, the images were input into the convolutional neural network model, and the classification results were combined to generate the final recognition results. Then, the loss value was calculated and the parameters were updated based on the results. The specific training process is shown in Eq. 2.


(2) $${\text{P}}_{\text{img}}=\text{S}\left(\sum_{\text{i}}^{\text{N}=2,4,8}{\text{P}}_{\text{i}}\right)$$

where P_img_ refers to the final classification results; P_i_ refers to the classification results at different granularities; S(⋅) refers the softmax function. Since there were disease spots at different granularities in the training process, the location of disease spot was not fixed. Therefore, the model would be able to better adapt to complex and diverse disease images after the training process, so as to improve the robustness of the model.

#### Graph Structure Branch

In order to better represent the relationship among disease attributes, feature words were extracted from the sequential text data and were recombined to form the graph structure data. Let G = {V,E} be a given graph dataset, where V refers to the node set andE refers to the edge set. Based on G, the adjacency matrix A and the degree matrix D can be obtained, and eventually, the feature matrix of the graph data can be obtained. Specifically, the feature matrix after the first layer of graph convolution L^(1)^ can be derived through Eq. 3, and the feature matrix after the second layer of graph convolution L^(2)^ can be derived through Eq. 4. Considering that the GCN might encounter the phenomenon of gradient disappearance or gradient explosion after multi-layer operations and in view of the characteristics of the disease dataset itself, only two graph convolution operations were performed in this study.


(3) $${\text{L}}^{\left(1\right)}=\rho \left(\tilde{\text{A}}{\text{XW}}_{0}\right)$$

(4) $${\text{L}}^{\left(2\right)}=\rho \left(\tilde{\text{A}}{\text{L}}^{\left(1\right)}{\text{W}}_{1}\right)$$

where $\tilde{\text{A}}={\text{D}}^{-\frac{1}{2}}{\text{AD}}^{-\frac{1}{2}}$; W_0_ and W_1_ refer to the weight matrix; ρ(⋅) refers to the activation function. In this study, the ReLU function was used. After the two-layer graph convolution operation, the classification results of node data were obtained. The classification process is shown in Eq. 5.


(5) $${\text{P}}_{\text{graph}}=\text{S}\left(\tilde{\text{A}}\rho \left(\tilde{\text{A}}{\text{XW}}_{0}\right){\text{W}}_{1}\right).$$

Finally, the recognition results of the image branch and the graph structure branch were fused, making sure that the recognition process not only learned the visual features of the disease image, but also incorporated the visual features of the disease in text description. The specific classification process is shown in Eq. 6. Both loss functions of image branch and graph structure branch are cross entropy loss functions, as shown in Eq. 7.


(6) $$\text{P}={\text{P}}_{\text{img}}+{\text{P}}_{\text{graph}}$$

(7) $$\text{Loss}=-\sum_{\text{i}=1,j=1}^{\text{T}}{\text{y}}_{\text{i}}^{\mathrm{img}/\mathrm{graph}}{\text{logP}}_{\text{j}}^{\mathrm{img}/\mathrm{graph}}$$

## Experiment

Both the study experiment and control experiment were carried out in the Ubuntu 18.04 environment: processor Intel core i9 9820X; memory 64G; graphics card GeForce RTX 2080Ti 11G DDR6. The deep learning framework Pytorch, in combination with Cuda9.0, was used for training. The batch-size of the training set and the validation set during the experiment design and the control process was set to be 16 and 8, respectively, based on the number of network parameters. The number of iterations of all network models was set to 50. The learning rate for model training is set to 0.0001, and the optimizer uses Adam. In addition, all models in the image branch adopt their corresponding network structure, and the number of final output layer classes is modified to 6. In order to ensure the fairness of performance comparison, all models do not use pre-training models.

### Evaluation Indicators

The models were compared from four aspects: recognition accuracy, recognition precision, sensitivity, and specificity. See Eqs 8–11 for the corresponding formulas.


(8) $$\text{Accuracy}=\frac{\text{TP}+\text{TN}}{\text{TP}+\text{TN}+\text{FP}+\text{FN}}\times 100\%$$

(9) $$\text{Precision}=\frac{\text{TP}}{\text{TP}+\text{FP}}\times 100\%$$

(10) $$\text{Sensitivity}=\frac{\text{TP}}{\text{TP}+\text{FN}}\times 100\%$$

(11) $$\text{Specificity}=\frac{\text{TN}}{\text{FP}+\text{TN}}\times 100\%$$

where TP refers to the number of samples belonging to category C and were correctly classified by the classifier; FP refers to the number of samples not belonging to category C but were misclassified by the classifier as category C; TN refers to the number of samples not belonging to category C and were correctly classified by the classifier; FN refers to the number of samples belonging to category C but were misclassified by the classifier.

### Comparison of Models for the Image Branch

**FIGURE 5:**
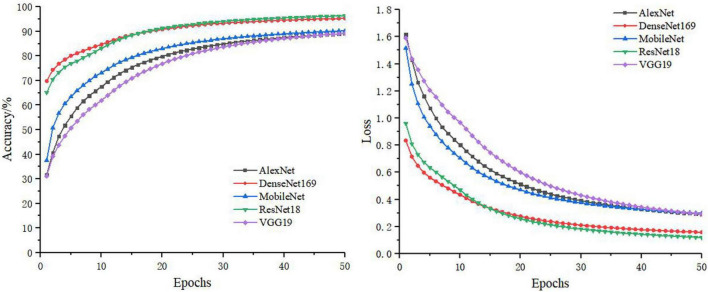
The training process using original images.

**FIGURE 6:**
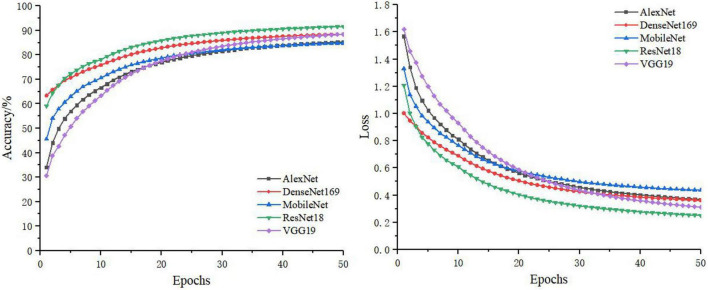
The training process with feature decomposition and recombination.

In the separate training process of the convolutional neural network, the selected control networks were AlexNet, ResNet18, DenseNet169, MobileNet, and VGG19. The training process without feature decomposition and recombination of the original image is shown in Figure 5, and the training process with feature decomposition and recombination is shown in Figure 6. In order to validate whether the feature decomposition and recombination method could improve the robustness of the model, two image datasets (i.e., one dataset with the same disease severity and one dataset with different disease severities) were used respectively for testing. The comparisons of testing results on the two datasets are shown in Tables 2, 3 respectively.

**TABLE 2:** Test results on the dataset with the same disease severity.

	Original images				Images with feature decomposition and recombination			
		
	Acc/%	Pre/%	Sen/%	Spe/%	Acc/%	Pre/%	Sen/%	Spe/%
AlexNet	93.77	81.25	81.12	96.28	90.84	73.61	71.79	94.55
**DenseNet169**	**95.05**	**86.54**	**85.03**	**97.05**	94.14	83.27	82.22	96.52
MobileNet	91.03	74.31	72.39	94.55	92.49	76.79	76.52	95.49
**ResNet18**	94.14	83.72	81.77	96.46	**95.42**	**87.50**	**85.63**	**97.27**
VGG19	89.74	67.93	68.24	93.87	89.56	67.70	67.28	93.71

*Bold represents that the comprehensive performance metrics of the model or scheme is the best.*

**TABLE 3:** Test results on the dataset with different disease severities.

	Original images				Images with feature decomposition and recombination			
		
	Acc/%	Pre/%	Sen/%	Spe/%	Acc/%	Pre/%	Sen/%	Spe/%
AlexNet	87.07	65.68	66.44	92.21	87.87	68.66	67.93	92.64
DenseNet169	87.55	64.32	65.98	92.58	85.70	60.31	64.50	91.34
MobileNet	86.10	55.82	60.83	91.78	86.51	61.41	62.41	91.92
**ResNet18**	**88.51**	**65.33**	**68.13**	**93.11**	**89.32**	**71.71**	**70.42**	**93.55**
VGG19	82.41	54.56	52.16	89.41	82.89	52.94	52.48	89.78

*Bold represents that the comprehensive performance metrics of the model or scheme is the best.*

During the training process, the images would become more complex after feature decomposition and recombination, and depending on the segmentation granularity, the same disease spot might be segmented into different blocks. Thus, the overall training burden was increased. Therefore, it can be seen from Figures 5, 6 that the model trained by original images achieved higher accuracy and lower loss. However, according to the test results on two different datasets, it was found that, although the model trained by original images performed better on the dataset with the same disease severity, but on the dataset with different disease severities, most of the models trained with feature decomposition and recombination achieved better outcomes. This proves that the feature decomposition and recombination method can contribute to the robustness of the model.

In the image branch, the feature decomposition and recombination method was used for model training. In order to identify the impact of different segmentation granularities on the recognition results, ResNet18 was chosen as the basic feature extraction network, and the granularity was set to 2, 4, 8, and the superimposition of the three (i.e., 2 + 4 + 8). The two datasets as mentioned earlier were used for testing, and the test results are shown in Table 4.

**TABLE 4:** Comparison of different segmentation granularities.

Split granularity	Test datasets with the same disease severity				Test datasets with the different disease severity			
	Acc/%	Pre/%	Sen/%	Spe/%	Acc/%	Pre/%	Sen/%	Spe/%
2	95.05	86.86	84.61	97.05	84.98	56.54	60.86	91.04
4	94.14	82.94	82.05	96.53	85.86	61.44	62.47	91.63
8	93.04	81.94	79.16	95.87	83.69	55.72	56.25	90.48
**2 + 4 + 8**	**95.42**	**87.50**	**85.63**	**97.27**	**89.32**	**71.71**	**70.42**	**93.55**

*Bold represents that the comprehensive performance metrics of the model or scheme is the best.*

**FIGURE 7:**
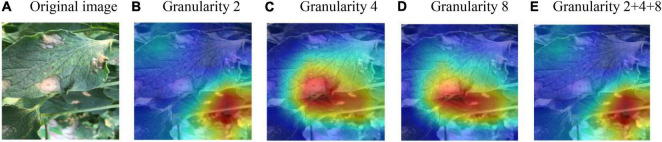
Visualization of the model’s region of interest. **(A)** Original image. **(B)** Granularity 2. **(C)** Granularity 4. **(D)** Granularity 8. **(E)** Granularity 2 + 4 + 8.

It can be seen from Table 4 that, on the test set with the same disease severity, granularity 2 achieved the best outcome, whereas on the test set with different disease severities, granularity 4 achieved the best outcome. With feature decomposition and recombination, the complexity of the image would increase with the increase of the segmentation granularity. As a result, large diseased areas might be randomly divided into any position of the image, which increased the training difficulty when using the dataset with the same disease severity. But on the dataset with different disease severities, the recognition outcomes appeared to be different. If the segmentation granularity was reasonable, the performance of the model would be improved as the segmentation granularity increased. Moreover, with the superimposition of different granularities (2 + 4 + 8), the model could achieve a better outcome than the optimal granularity. In order to observe the regions of interest of the model on the disease image, Grad-cam++ (Chattopadhay et al., 2018) was used to visualize the model, and the results are shown in Figure 7.

It can be seen from Figure 7 that the models trained at all granularities could accurately recognize the diseased area. Models trained at granularity 4 and 8 had similar regions of interest, while models trained at granularity 2 and 2 + 4 + 8 had similar regions of interest. The model trained at a larger granularity was more sensitive to larger diseased areas, whereas the model trained at a smaller granularity was more sensitive to smaller diseased areas. Furthermore, with the superimposition of different granularities, the model was more likely to be affected by the small-granularity segmentation model. By comprehensively considering the recognition accuracy of different models on the test set and the model’s regions of interest, it was found that the models trained at different granularities had their respective advantages. Therefore, in this study, the feature segmentation for the image branch integrated different granularities in order to achieve accurate acquisition and learning on the diseased area.

### Comparison of Models for the Graph Structure Branch

**FIGURE 8:**
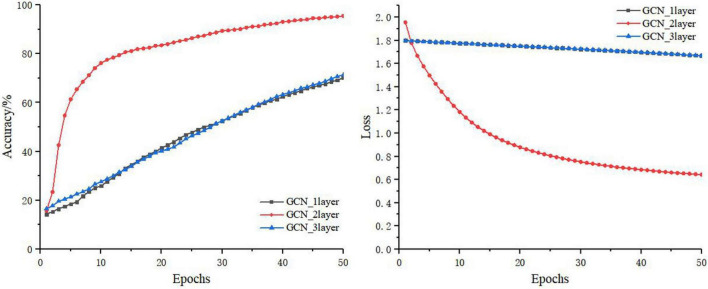
The GCN training process for different layers.

The GCN takes the features of the current node itself and the relationship between the current node and its neighbors as the network training parameters. The features of the current node are always updated based on the features of the previous node. Thus, the number of layers of the graph neural network (i.e., the number of hops in the neighbors of the node) is very important to the final outcome of the model. In this section, the number of layers of the GCN was set to 1, 2, and 3, respectively. The training process is shown in Figure 8, and the test results of the test set are shown in Table 5.

**TABLE 5:** The GCN test results for different layers.

	Accuracy/%	Precision/%	Sensitivity/%
GCN_1layer	45.60	45.38	45.62
GCN_2layer	82.42	82.83	82.47
GCN_3layer	43.96	42.46	43.22

It can be seen from Figure 8 and Table 5 that, on the basis of the same number of training iterations, the two-layer GCN model was significantly advantageous to the one-layer and three-layer GCN models in terms of accuracy, precision, sensitivity, and loss. Because in the graph structure dataset based on disease knowledge, most of the disease information is directly related to the disease category, its adjacency matrix has therefore a larger weight. In addition, Kip and Welling (2016) and Li et al. (2018) also reported that a GCN that was too deep would lead to the problems of gradient disappearance or gradient explosion, while a GCN that was too shallow would lead to poor performance due to fewer learning features. Therefore in this study, the two-layer GCN was chosen for knowledge supplementation and fusion with the convolutional neural network.

**FIGURE 9:**
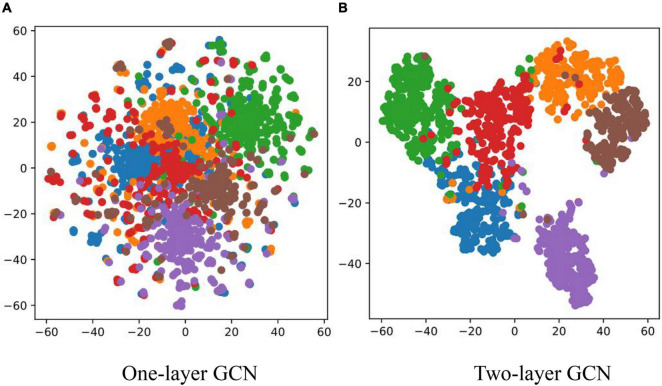
Clustering results of text nodes. **(A)** One-layer GCN. **(B)** Two-layer GCN.

In order to demonstrate the effectiveness of the graph structure text in the process of disease recognition and to further explain the basis of judgments, this section classified the diseases according to word nodes and text nodes, and then, the top five words in the word node with the highest recognition confidence were extracted. The words with the highest confidence among different categories are summarized in Table 6. In the text node, different text nodes were clustered according to the final node representation of the model and clustering method is T-distributed Stochastic Neighbor Embedding (t-SNE). The clustering results are shown in Figure 9.

**TABLE 6:** Disease feature words.

Tomato powdery mildew	Tomato early blight	Cucumber powdery mildew	Cucumber virus disease	Cucumber downy mildew	Cucumber bacterial spot
Powdery	Blobs	Protrusions	Watery	Polygonal	Gray
White	Tomato	Raised	Beans	Square	Radiating
Spotty	Obverse	Inward	Petiole	Regular	Hair
Melatonin	Brown	Pattern	Sized	Dark	Evenly
Middle	Ring	Shrink	Sporadic	Rectangular	Sides

It can be seen from Table 6 that most of the five feature words with the highest correlation to each disease category that were obtained by the GCN could correctly represent the feature of the corresponding disease category, but there were also some non-feature words with weak correlation. However, in general, the graph structure text could provide knowledge information for the disease image recognition process, and guide the model training to a certain extent. Figure 9 compares the effects of one-layer GCN and two-layer GCN in text node clustering. It can be seen that the clustering effect of two-layer GCN was significantly advantageous to that of one-layer GCN. This is consistent with the recognition outcomes as shown in Table 5.

### Comparison of Fusion Models

In the image branch, the feature decomposition and recombination method was applied study to improve the robustness of the model, but the actual application effect differed for different choices of the basic network structure. On the test set with the same disease severity, DenseNet169 achieved the best recognition accuracy, but on the test set with different disease severities, ResNet18 achieved the best performance. Moreover, in both test sets, the performance of ResNet18 was improved after applying the feature decomposition and recombination method. Therefore, in this study, ResNet18 was chosen as the convolutional neural network. In the graph structure branch, as the two-layer GCN appeared to be more suitable than the one-layer and three-layer networks for the graph structure based on disease knowledge, the two-layer GCN was used. The test results of the fusion model are shown in Table 7.

**TABLE 7:** Test results of the fusion model.

	Accuracy/%	Precision/%	Sensitivity/%	Specificity/%
Tomato powdery mildew	97.25	86.67	99.34	96.30
Tomato early blight	100	100	100	100
Cucumber powdery mildew	95.60	91.67	96.58	86.84
Cucumber virus disease	98.35	96.55	98.69	93.33
Cucumber downy mildew	96.70	94.44	97.26	89.47
Cucumber bacterial spot	97.80	87.50	99.37	95.45
**Average**	**97.62**	**92.81**	**98.54**	**93.57**

*The bold terms and values represent the average values of the performance metrics of model recognition on the data of different disease categories.*

According to Tables 2, 5, 7, the accuracy, precision, sensitivity and specificity of the fusion model were improved to varying degrees for all disease categories. In terms of accuracy, the fusion model was improved by about 3% compared with the original ResNet18 model, and by about 15% compared with the two-layer graph neural network. Therefore, it can be concluded that the fusion model not only learned the visual features in the image, but also made corrections on the recognition results according to the non-image features in the graph structure. As a result, it achieved the best outcome.

## Discussion

Aiming at the problem of weak robustness of conventional feature extraction networks to datasets with different disease severities, a feature decomposition and recombination method was proposed in this study, which improved the robustness of the original feature extraction network to a certain extent. However, the effect of this method differed for different network structures. Generally speaking, it could derive an ideal model on the dataset with the same disease severity. On the dataset with different disease severities, its performance still maintained at a high level, though showing a certain degree of decrease. Therefore, the future research should consider how to improve the robustness of feature extraction models with different structures. In view of that conventional disease recognition methods lack the use of other expressions of disease visual factors, this study proposed to use GCN to train the visual disease text description information, and a graph structure disease dataset was built. However, this dataset is static. With the continuous increase of disease information, new datasets need to be built in the future. Thus, the follow-up research should consider using the dynamic graph neural network training method for optimization.

## Conclusion

Conventional disease recognition methods lack the use of modal information other than the image modality. In the present study, the disease text description information represented by continuous vectors was decomposed and recombined into graph structure data. For image data, the feature decomposition was implemented by randomly disarranging and recombining the image blocks after segmentation, which improved the robustness of the model to a certain extent. Specifically, the accuracy, precision, sensitivity and specificity of the fusion model were 97.62, 92.81, 98.54, and 93.57%, respectively. This research provides new ideas for disease recognition, and puts forward new insights and methodology in improving the robustness of disease recognition models.

## Data Availability Statement

The original contributions presented in the study are included in the article/supplementary material, further inquiries can be directed to the corresponding authors.

## Author Contributions

CW: writing original draft. JZ: software and validation. YZ: data curation. HW: methodology, and writing – review and editing. CZ: writing – review and editing, and supervision. GT: investigation. JL: visualization. All authors contributed to the article and approved the submitted version.

## Conflict of Interest

The authors declare that the research was conducted in the absence of any commercial or financial relationships that could be construed as a potential conflict of interest.

## Publisher’s Note

All claims expressed in this article are solely those of the authors and do not necessarily represent those of their affiliated organizations, or those of the publisher, the editors and the reviewers. Any product that may be evaluated in this article, or claim that may be made by its manufacturer, is not guaranteed or endorsed by the publisher.
